# A Casein Kinase 1 and PAR Proteins Regulate Asymmetry of a PIP_2_ Synthesis Enzyme for Asymmetric Spindle Positioning

**DOI:** 10.1016/j.devcel.2008.06.002

**Published:** 2008-08-12

**Authors:** Costanza Panbianco, David Weinkove, Esther Zanin, David Jones, Nullin Divecha, Monica Gotta, Julie Ahringer

**Affiliations:** 1 The Gurdon Institute and Department of Genetics, University of Cambridge, Tennis Court Road, Cambridge CB2 1QN, UK; 2 Division of Cellular Biochemistry, The Netherlands Cancer Institute, Plesmanlaan 121, 1066 CX Amsterdam, The Netherlands; 3 The Inositide Laboratory, The Paterson Institute for Cancer Research, University of Manchester, Manchester M20 4BX, UK; 4 Institute of Biochemistry, Building HPM, Room G16.2, ETH Hoenggerberg, 8093-Zuerich, Switzerland

**Keywords:** DEVBIO, CELLBIO

## Abstract

Spindle positioning is an essential feature of asymmetric cell division. The conserved PAR proteins together with heterotrimeric G proteins control spindle positioning in animal cells, but how these are linked is not known. In *C. elegans*, PAR protein activity leads to asymmetric spindle placement through cortical asymmetry of Gα regulators GPR-1/2. Here, we establish that the casein kinase 1 gamma CSNK-1 and a PIP_2_ synthesis enzyme (PPK-1) transduce PAR polarity to asymmetric Gα regulation. PPK-1 is posteriorly enriched in the one-celled embryo through PAR and CSNK-1 activities. Loss of CSNK-1 causes uniformly high PPK-1 levels, high symmetric cortical levels of GPR-1/2 and LIN-5, and increased spindle pulling forces. In contrast, knockdown of *ppk-1* leads to low GPR-1/2 levels and decreased spindle forces. Furthermore, loss of CSNK-1 leads to increased levels of PIP_2_. We propose that asymmetric generation of PIP_2_ by PPK-1 directs the posterior enrichment of GPR-1/2 and LIN-5, leading to posterior spindle displacement.

## Introduction

Asymmetric cell divisions are important for the fate and diversity of many animal cells (reviewed in Betschinger and Knoblich, 2004). To ensure the proper inheritance of localized molecules, the position and orientation of the mitotic spindle must be coupled to overall cell polarity. A wealth of studies from many systems has shown that the molecules involved in cell polarization and spindle positioning are similar in different animal cells, suggesting the existence of a universal mechanism that has been conserved throughout evolution. PAR polarity proteins are used for polarization, and they control spindle position through regulation of heterotrimeric G protein signaling (reviewed in McCarthy and Goldstein, 2006). However, the mechanism of this coupling is not understood.

The *C. elegans* embryo is an important model for studying asymmetric cell division (reviewed in Schneider and Bowerman, 2003). The *par* genes, key polarity regulators in animal cells, were initially identified in the worm through the identification of mutants that disrupt cell polarity at the one-celled stage (Kemphues et al., 1988). Many of the PAR proteins show asymmetric protein localization. In the one-celled *C. elegans* embryo, a complex of PAR-3 and PAR-6, two PDZ-domain-containing proteins, together with atypical protein kinase C PKC-3 are found at the anterior. PAR-1, a ser/thr kinase, and PAR-2, a RING finger domain protein, are found at the posterior (reviewed in Cowan and Hyman, 2007). The PAR proteins control downstream cortical and cytoplasmic protein asymmetries. For asymmetric spindle positioning, PAR polarity is translated into asymmetric spindle pulling forces, with strong forces acting on the posterior aster and weak forces acting on the anterior aster (Grill et al., 2001, 2003). The precise mechanism by which the PAR proteins coordinate polarity with spindle positioning remains to be elucidated; however, G protein signaling has been identified as the major spindle force transducer.

In *C. elegans*, two partially redundant Gα subunits (GOA-1 and GPA-16) together with their receptor-independent activators GPR-1 and GPR-2 (GPR-1/2) and the coil-coiled protein LIN-5 are required for asymmetric spindle positioning and overall pulling forces; inactivation of any of these proteins results in strongly reduced pulling forces and symmetric placement of the first spindle (Colombo et al., 2003; Gotta and Ahringer, 2001; Gotta et al., 2003; Grill et al., 2003; Lorson et al., 2000; Miller and Rand, 2000; Srinivasan et al., 2003; Tsou et al., 2003). The Gα subunits, GPR-1/2, and LIN-5 form a complex that regulates dynein-mediated pulling forces (Afshar et al., 2005; Colombo et al., 2003; Couwenbergs et al., 2007; Gotta et al., 2003; Nguyen-Ngoc et al., 2007; Srinivasan et al., 2003). Whereas Gα subunits are uniformly distributed, both GPR-1/2 and LIN-5 are posteriorly enriched (Colombo et al., 2003; Gotta et al., 2003; Park and Rose, 2008; Tsou et al., 2003). This asymmetry of GPR-1/2 and LIN-5 is controlled by the PAR proteins (Colombo et al., 2003; Gotta et al., 2003; Park and Rose, 2008; Srinivasan et al., 2003). Therefore, cortical PAR polarity is transduced to asymmetric spindle positioning through asymmetric G protein regulation, but the mechanism of this coupling is unknown.

The crucial role of G protein signaling in spindle orientation and/or positioning is conserved in other animals. Pins, the *Drosophila* homolog of GPR-1/2, shows asymmetric cortical localization dependent on PAR proteins and plays a key role in neuroblast asymmetric cell division. Pins binds and functions together with Gα subunits and the protein Mud, thought to be the functional homolog of LIN-5 (Schaefer et al., 2001; Yu et al., 2000). Similarly, in mammals, the GPR-1/2 homolog LGN, together with Gα subunits and NuMA (similar to *Drosophila* Mud), regulates the spindle through associations with dynein/dynactin (Bowman et al., 2006; Du and Macara, 2004; Hampoelz and Knoblich, 2004; Haren and Merdes, 2002; Izumi et al., 2006; Siller et al., 2006). Ags3, another GPR-1/2 homolog, controls spindle orientation in the mammalian brain together with Gα subunits (Sanada and Tsai, 2005).

In *C. elegans*, PAR proteins control the asymmetry of forces acting on the spindle by inducing asymmetry of cortical GPR-1/2, but how this information is transduced remains to be elucidated. To identify new genes involved in this process, we previously carried out an RNAi time-lapse video recording screen, where we identified a gamma isoform of casein kinase 1, *csnk-1/*Y106G6E.6, as a candidate (Zipperlen et al., 2001). Here we demonstrate a role for CSNK-1 in linking PAR polarity to the regulation of GPR-1/2 and mitotic spindle pulling forces via the asymmetric enrichment of the PtdIns(4,5)P_2_ (PIP_2_) generating enzyme PPK-1.

## Results

### Knockdown of CSNK-1 Results in Aberrant Spindle Positioning

**Figure 1:**
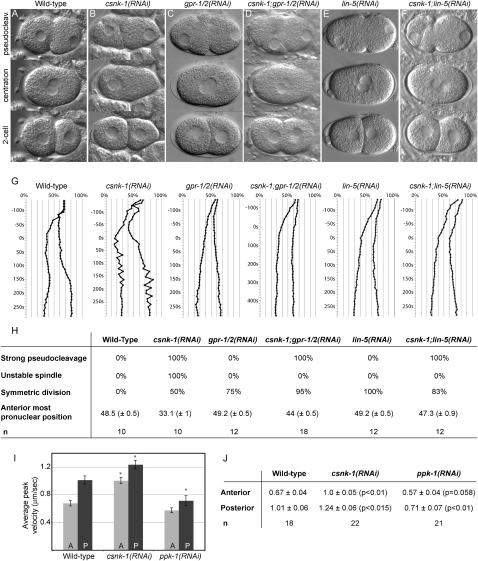
Excessive Pronuclear and Spindle Movements in *csnk-1(RNAi)* Embryos Depend on GPR-1/2 and LIN-5 (A–F) Pseudocleavage, centration (just prior to nuclear envelope breakdown), and two-cell stage images taken from time-lapse DIC video recordings in the indicated backgrounds. First row: abnormally strong pseudocleavage is observed in (B) *csnk-1(RNAi)*, (D) *csnk-1;gpr-1/2(RNAi)*, and (F) *csnk-1;lin-5(RNAi)* compared to (A) wild-type. Second row: pronuclear position at nuclear envelope breakdown (NEBD) in (B) *csnk-1(RNAi)* embryo is anterior compared to other backgrounds. Third row: (A) a wild-type embryo underwent asymmetric cell division (larger anterior cell), whereas embryos of other backgrounds have two equal sized cells. (G) Traces of anterior and posterior centrosome positions in representative one-cell embryos from pronuclear meeting to cytokinesis onset in the indicated backgrounds. 0% and 100% represent anterior and posterior ends, respectively. Time 0 s indicates NEBD. Note the large and rapid movements of the centrosomes in *csnk-1(RNAi)* compared to the other backgrounds. (H) Quantification of the phenotypes described in (A)–(F). Asymmetric division is defined as 52%–56% egg length, symmetric division as 48%–52%. In the anterior-most pronuclear position, 0 and 100 represent anterior and posterior ends, respectively. n, number of embryos analyzed. In this and other figures anterior is to the left and posterior to the right. (I and J) A graph (I) and table (J) show mean peak velocities (micrometer/second) of anterior (light gray) and posterior (dark gray) spindle poles measured after spindle severing in one-cell embryos of indicated genotypes. Error bars correspond to SEM. ^∗^p < 0.05 compared to corresponding wild-type. Exact p values are given in the table. n, number of embryos analyzed.

To investigate potential roles for CSNK-1 in spindle positioning, we carried out a detailed analysis of the first cell division using time-lapse videomicroscopy. For comparison of the defects, we review here the major events in wild-type. After fertilization and completion of meiosis I and II, the maternal and paternal pronuclei are usually at opposite ends of the oval embryo. The female pronucleus migrates toward its paternal partner, during which time a centrally located pseudocleavage furrow is formed (Figure 1A, pseudocleavage). The pronuclei meet at the posterior, and then the complex moves to the center while rotating 90° to align the centrosomes along the anterior posterior axis (Figure 1A, centration). The first spindle sets up centrally but is pulled toward the posterior, culminating in an asymmetric first cell division with a larger anterior cell, AB, and a smaller posterior cell, P1 (Figure 1A, 2-cell).

*csnk-1(RNAi)* embryos show cortical defects and abnormalities in pronuclear and spindle positioning. First, the pseudocleavage furrow is unusually deep and prolonged (Figures 1B and 1H). Second, after pronuclear meeting the pronuclear-centrosomal complex is invariably pulled to the anterior of the embryo rather than moving to the center (Figures 1B and 1H). Third, the nuclear envelope breaks down, and mitotic spindle assembly takes place anterior to the normal location (data not shown). Fourth, spindle position is extremely unstable, with the spindle displaying exaggerated rocking movements resulting in a symmetric first division in about 50% of embryos (Figures 1B and 1H).

To analyze *csnk-1(RNAi)* centrosome and spindle pole movements in more detail, we tracked the position of each centrosome from pronuclear meeting until the onset of cytokinesis (Figure 1G). Whereas wild-type plots show smooth movements throughout this period, centrosome position plots of *csnk-1(RNAi)* embryos are jagged. There is extreme aberrant movement of centrosome positions as the pronuclear complex moves toward the anterior, and after the spindle has formed (Figure 1G). These excessive movements suggest a role for CSNK-1 in negative regulation of pronuclear and spindle pulling forces.

To test this hypothesis, we compared spindle pulling forces in *csnk-1(RNAi)* embryos to those in wild-type. After severing the metaphase mitotic spindle with a laser microbeam, the peak anterior and posterior velocities of the independent spindle poles are a readout of the net pulling forces acting on each side (Grill et al., 2001). As previously reported, wild-type spindle pulling forces are asymmetric, with lower anterior than posterior peak velocities after severing (Grill et al., 2001) (Figures 1I and 1J). We found that *csnk-1(RNAi)* embryos show significantly increased anterior and posterior spindle pole peak velocities compared to wild-type (Figures 1I and 1J). We conclude that CSNK-1 negatively regulates spindle pulling forces.

### *csnk-1(RNAi)* Embryos Show Increased Cortical GPR-1/2 and LIN-5

Because spindle pulling forces require regulation of Gα subunits through their receptor-independent activators GPR-1/2 and the LIN-5 protein, we considered that they might be abnormally regulated in *csnk-1(RNAi)* embryos. We first tested whether the abnormal pronuclear and spindle movements depended on GPR-1/2 and LIN-5. Indeed, we found that *csnk-1(RNAi);gpr-1/2(RNAi)* and *csnk-1(RNAi);lin-5(RNAi)* embryos both lack the jerky movements of *csnk-1(RNAi)* embryos and instead look like *gpr-1/2(RNAi)* or *lin-5(RNAi)* embryos alone (Figures 1C–1H). In contrast, the strong pseudocleavage furrow defect of *csnk-1(RNAi)* embryos is not rescued (Figure 1C–1F and 1H). We conclude that the excessive spindle movements of *csnk-1(RNAi)* embryos are mediated by Gα/GPR-1/2/LIN-5 activity.

**Figure 2:**
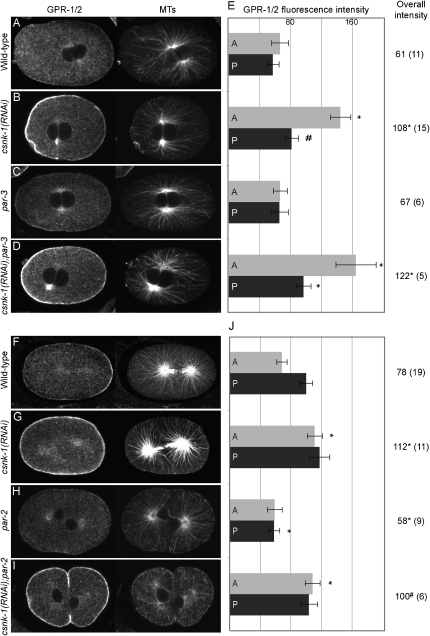
CSNK-1 Acts Downstream of PAR-2 and PAR-3 for Control of GPR-1/2 Distribution and Spindle Movements GPR-1/2 (left panels) and tubulin (right panels) staining in embryos of indicated genotypes. (A–D) One-cell embryos at pronuclear meeting. (F–I) One-cell embryos at ana-telophase. (E and J) Quantification of average anterior cortical GPR-1/2 pixel intensities (0%–25% egg length; light gray) and posterior cortical GPR-1/2 pixel intensities (75%–100% egg length; dark gray) in embryos of genotypes indicated at the left. Error bars represent SEM. ^∗^p < 0.05; ^#^p < 0.056 compared to corresponding wild-type. Overall intensity signifies the average intensity of the whole embryonic cortex. Numbers in brackets show the number of embryos analyzed. In 3/3 *csnk-1(RNAi)* embryos, pronuclei move anterior to 40% egg length during centration compared to 0/10 for wild-type, 0/4 for *par-2* mutants, and 0/5 for *par-3* mutants. In 7/7 *csnk-1(RNAi);par-2* embryos and 13/17 *csnk-1(RNAi);par-3* embryos, pronuclei moved anterior to 40% egg length, similar to *csnk-1(RNAi)* embryos. In 3/3 *csnk-1(RNAi)* embryos, the spindle showed jerky unstable movement (rapid spindle movement along both long and short axes) compared to 0/10 for wild-type, 0/4 for *par-2*, and 0/5 for *par-3* embryos. In 7/7 *csnk-1(RNAi);par-2* embryos and 17/17 *csnk-1(RNAi);par-3* embryos, the spindle showed unstable movements similar to *csnk-1(RNAi)* embryos.

To explore regulation of Gα/GPR-1/2/LIN-5 by CSNK-1 further, we examined the pattern of localization of these proteins in *csnk-1(RNAi)* embryos. We found that GOA-1 and GPA-16 show a normal distribution (see Figure S1 available online). In contrast, the patterns of GPR-1/2 and LIN-5 are significantly altered. In wild-type one-celled embryos, GPR-1/2 and LIN-5 show weak anterior cortical enrichment during pronuclear centration followed by stronger posterior enrichment from metaphase (Figures 2A, 2E, 2F, and 2J and Figure S3) (Colombo et al., 2003; Gotta et al., 2003; Park and Rose, 2008). This anterior enrichment plays a role in nuclear centration (Park and Rose, 2008).

We observed three defects in the pattern of GPR-1/2 localization in *csnk-1(RNAi)* embryos. First, GPR-1/2 has increased cortical association at all embryonic stages (Figures 2B, 2G, 2E and 2J, and data not shown). Because all embryos strongly stained for CSNK-1, we investigated whether oocytes also showed abnormalities. We found that *csnk-1(RNAi)* oocytes similarly have increased cortical staining of GPR-1/2 compared to wild-type (Figures S2A, S2B, and S2D). Second, during pronuclear meeting and centration, *csnk-1(RNAi)* embryos show a strong anterior enrichment of GPR-1/2 (Figures 2B and 2E). This corresponds to the time when pronuclei in *csnk-1(RNAi)* embryos are abnormally pulled to the anterior and to the time of weak anterior GPR-1/2 enrichment of wild-type embryos. Lastly, *csnk-1(RNAi)* embryos show no posterior enrichment of GPR-1/2 from metaphase to early two-cell stage embryos, in contrast to wild-type embryos (Figures 2G and 2J). Total GPR-1/2 protein levels, assayed by western blot analysis, are comparable in wild-type and *csnk-1(RNAi)* embryos, indicating that the increased cortical association is not due to increased protein levels (Figure S2E). This indicates that the normally cytoplasmic pool of GPR-1/2 is ectopically recruited to the cortex in the absence of CSNK-1. We found that the distributions of LIN-5 in *csnk-1(RNAi)* embryos show similar alterations (Figure S3).

We conclude that CSNK-1 negatively regulates cortical association of GPR-1/2 and LIN-5 during asymmetric spindle positioning and both are required for excessive pronuclear and spindle movements. As GPR-1/2 and LIN-5 are codependent on each other for their cortical localization (Gotta et al., 2003; Park and Rose, 2008; Srinivasan et al., 2003), we cannot distinguish whether one or both are targets of this regulation.

### CSNK-1 Localization

**Figure 3:**
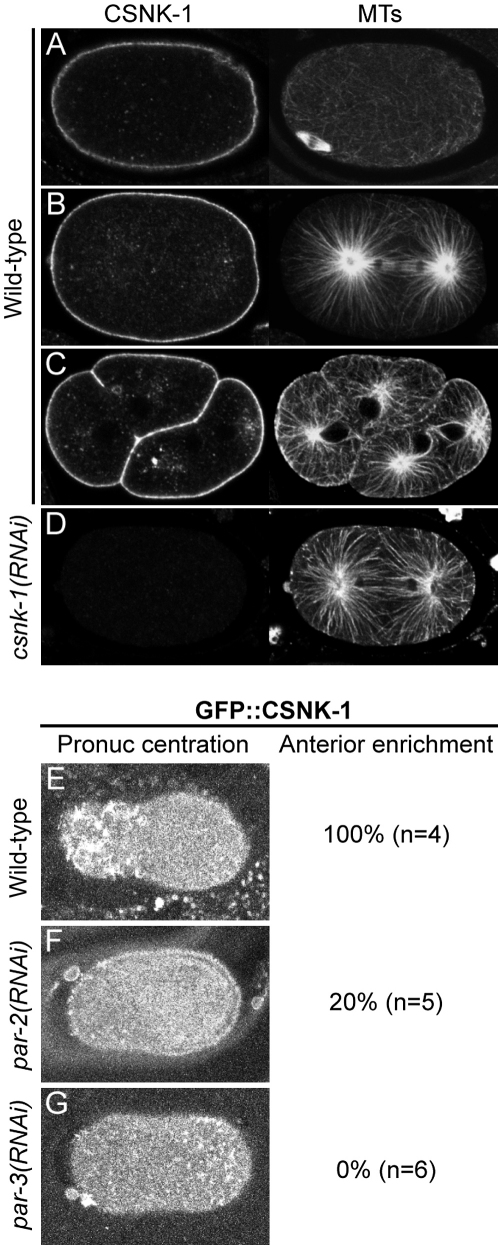
CSNK-1 Localization (A–D) Wild-type embryos stained for CSNK-1 (left panels) and tubulin (right panels). (A) meiotic embryo, (B) anaphase embryo, (C) four-cell embryo. Staining is specific, as it is absent from (D) *csnk-1(RNAi)* embryos. (E–G) Projection of two cortical sections of GFP::CSNK-1 at pronuclear centration in indicated genotypes; numbers at the right give the percentage of embryos showing anterior enrichment of GFP::CSNK-1. n, number of embryos analyzed.

To determine where CSNK-1 protein is localized, we raised an antibody against the C-terminal part of the protein. As predicted from the consensus palmityolation site at the C terminus, CSNK-1 is associated with the plasma membrane at all stages of the cell cycle (Figures 3A–3C). In addition, punctate staining is visible around the asters during mitosis (Figures 3B and 3C). This staining pattern corresponds to CSNK-1, as it is lost in *csnk-1(RNAi)* embryos (Figure 3D).

To investigate the dynamics of CSNK-1 protein localization, we constructed a functional GFP::CSNK-1 transgenic line (see the Experimental Procedures). As seen with the antibody, GFP::CSNK-1 localizes at the membrane at all stages. In addition, puncta form at the plasma membrane and move toward the asters (Movie S1). Before polarization, GFP::CSNK-1 appears as small foci and short filaments throughout the cortex (data not shown). Shortly after polarization, the GFP::CSNK-1 foci move away from the posterior cortex toward the anterior, resulting in an anterior cortical enrichment of the GFP::CSNK-1 foci (Figure 3E). This pattern of anterior cortical enrichment is similar to that seen for RHO-1 and the nonmuscle myosin NMY-2 (Motegi and Sugimoto, 2006; Munro et al., 2004; Schonegg and Hyman, 2006). Anterior enrichment appears to be sensitive to fixation, as it is not seen for CSNK-1 or GFP::CSNK-1 in fixed samples (data not shown). To summarize, CSNK-1 is found at the cortex and in cytoplasmic puncta, and a functional GFP tagged protein is anteriorly enriched.

### CSNK-1 Acts Downstream of PAR Polarity

Despite the fact that *csnk-1(RNAi)* embryos often have a symmetric first cleavage as in *par* polarity mutants, embryonic polarity appears to be normal, as PAR-2 and PAR-3 are correctly localized (Figure S4). This suggests that CSNK-1 regulates cortical forces downstream or in parallel to PAR proteins.

To investigate the relationship between the PAR proteins and CSNK-1, we performed genetic epistatic experiments. *csnk-1(RNAi)* embryos show anterior pronuclear movement during centration and jerky unstable spindle positioning, whereas in *par-2* and *par-3* mutant embryos, pronuclei move to the center and spindle movements are smooth (Figure 1G and Figure 2, legend). We found that both *csnk-1(RNAi);par-2* and *csnk-1(RNAi);par-3* mutant embryos show anterior pronuclear displacement and jerky spindle movements similar to those of *csnk-1(RNAi)* embryos (see Figure 2, legend). This suggests that CSNK-1 acts downstream or in parallel to PAR-2 and PAR-3.

We next examined the relationship between CSNK-1 and these PAR proteins in the regulation of GPR-1/2 localization. At pronuclear meeting, *par-3* mutant embryos show a weak symmetric localization of GPR-1/2 in contrast to the strong anterior enrichment of *csnk-1(RNAi)* embryos (Figures 2C and 2E) (Park and Rose, 2008). We found that *csnk-1(RNAi);par-3* embryos have strong anterior cortical GPR-1/2, similar to *csnk-1(RNAi)* embryos (Figures 2D and 2E). Therefore, *csnk-1* is epistatic to *par-3* for early GPR-1/2 localization. After metaphase, *par-3* mutant embryos show uniform high cortical GPR-1/2 levels similar to *csnk-1(RNAi)* embryos, and this is not further increased in *csnk-1(RNAi);par-3* embryos, suggesting that PAR-3 may positively regulate CSNK-1 at this time (Gotta et al., 2003) (Figure S5).

We found that *csnk-1* is also epistatic to *par-2* for GPR-1/2 localization. In *par-2* mutant embryos from metaphase to telophase, GPR-1/2 shows a symmetric distribution and lower overall levels compared to wild-type (Figures 2H and 2J). *csnk-1(RNAi);par-2* embryos show high symmetric GPR-1/2 similar to *csnk-1(RNAi)* embryos (Figures 2I and 2J). Thus, CSNK-1 acts downstream or in parallel to PAR-2.

To investigate the relationship between CSNK-1 and PAR polarity more directly, we asked whether the anterior localization of GFP::CSNK-1 depended on PAR-2 and/or PAR-3. Whereas wild-type embryos show anterior enrichment of GFP::CSNK-1 at pronuclear centration (Figure 3E), *par-2(RNAi)* and *par-3(RNAi)* embryos show symmetric distributions (Figures 3F and 3G, respectively). The requirement for PAR-2 and PAR-3 in CSNK-1 asymmetry together with the epistasis experiments indicates that CSNK-1 acts downstream of PAR polarity.

### The PIP_2_ Synthesis Enzyme PPK-1 Is Posteriorly Enriched

How might CSNK-1 regulate LIN-5 and GPR-1/2 localization and spindle forces? In budding yeast, there are two orthologs of CSNK-1, the functionally redundant genes yck-1/2 (Robinson et al., 1992; Wang et al., 1992). A direct target is MSS4, a PI(4)P5-kinase that converts PtdIns(4)P to PtdIns(4,5)P_2_, or PIP_2_ (Audhya and Emr, 2003). PPK-1 is the sole *C. elegans* ortholog of MSS4. To investigate whether PPK-1 might be relevant for spindle positioning in *C. elegans*, we first examined its localization by immunofluorescence.

**Figure 4:**
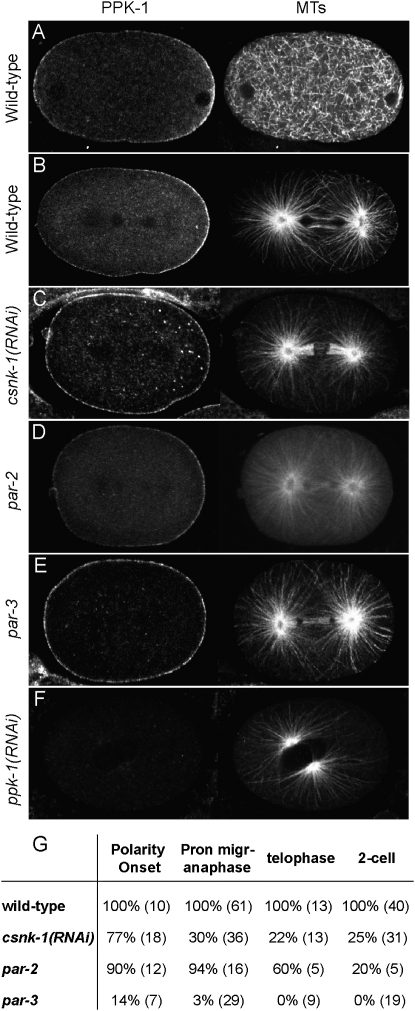
Posterior Enrichment of PPK-1 Is Controlled by CSNK-1, PAR-2, and PAR-3 Wild-type (A and B), *csnk-1(RNAi)* (C), *par-2* (D), *par-3* (E), and *ppk-1(RNAi)* (F) embryos stained for PPK-1 (left panels) and tubulin (right panels). (A) One-cell embryo at polarity onset. (B–E) One-cell embryos at anaphase. (F) One-cell embryo at pronuclear centration. PPK-1 is enriched at the posterior in wild-type (A and B) and *par-2* (D) embryos. PPK-1 shows symmetric distribution in *csnk-1(RNAi)* (C) and *par-3* (E) embryos. Staining is specific, as it is absent from *ppk-1(RNAi)* embryos (F). (G) Percent of embryos showing posterior PPK-1 enrichment in indicated backgrounds. Numbers in brackets show the number of embryos analyzed.

Strikingly, we found that PPK-1 is enriched at the posterior of the one-celled embryo (Figures 4A and 4B). Asymmetry of PPK-1 is first detectable around the time of polarity induction (Figure 4A). Often a small transient anterior cap is also observable (Figure 4A). PPK-1 is enriched at the posterior of the embryo until the four-cell stage, after which PPK-1 localization has not been analyzed. The asymmetric localization corresponds to PPK-1, as it is lost in *ppk-1(RNAi)* embryos (Figure 4F). Because PI(4)P5-kinases are PIP_2_-generating enzymes, these results suggest that PIP_2_ levels may be asymmetric at the membrane of the one-celled *C. elegans* embryo.

### PPK-1 Asymmetry Is Regulated by PAR-3, PAR-2, and CSNK-1

To test whether PPK-1 asymmetry is regulated by PAR polarity, we looked at its localization in *par-3* and *par-2* mutant embryos. We found that PPK-1 asymmetry depends on PAR-3 at all stages (Figures 4E and 4G). In contrast, PPK-1 asymmetry is established, but not maintained, in *par-2* mutant embryos (Figures 4D and 4G). The late requirement for PAR-2 probably reflects the role of PAR-2 in the maintenance, but not the establishment, of PAR-3 asymmetry (Cuenca et al., 2003).

We next investigated whether CSNK-1 regulates PPK-1 asymmetry. Most early *csnk-1(RNAi)* embryos showed PPK-1 asymmetry, but this was lost from pronuclear migration onward, where instead a symmetric distribution was observed (Figures 4C and 4G). This coincides with the initiation of anterior GFP::CSNK-1 enrichment. The increase in PPK-1 at the anterior cortex in *csnk-1(RNAi)* embryos suggests that CSNK-1 negatively regulates PPK-1. We conclude that PPK-1 asymmetry depends on CSNK-1 and on the establishment of PAR polarity.

### PPK-1 Regulates GPR-1/2 Localization and Spindle Movements

**Figure 5:**
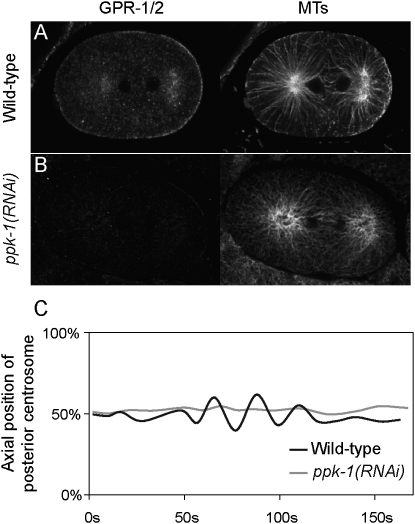
Reduced GPR-1/2 and Reduced Spindle Rocking in *ppk-1(RNAi)* Embryos Wild-type (A) and *ppk-1(RNAi)* (B) telophase embryos stained for GPR-1/2 (left panels) and tubulin (right panels). GPR-1/2 staining is highly reduced in 90% of *ppk-1(RNAi)* embryos (n = 10). (C) Posterior centrosome position in the short axis of the egg from metaphase (0 s) to cytokinesis (150 s) in representative wild-type (black line) and *ppk-1(RNAi)* embryos (gray line). Percentage represents percentage of egg width. *ppk-1(RNAi)* shows reduced posterior spindle pole rocking compared to wild-type (7.1% average width in *ppk-1(RNAi)* embryos [n = 6] versus 18.4% in wild-type [n = 5]).

If CSNK-1 acts negatively on PPK-1 for the regulation of spindle movements, then loss of PPK-1 should cause reduced GPR-1/2 localization and reduced spindle movements. Because strong knockdown of *ppk-1* by RNAi leads to sterility in the adult hermaphrodite (Xu et al., 2007), we could not analyze embryos completely depleted of PPK-1. We therefore used weaker RNAi conditions to obtain embryos with a partial loss of PPK-1. In these embryos, PPK-1 levels are strongly reduced (Figure 4F). Like in *csnk-1(RNAi)* embryos, we found that GOA-1 and GPA-16 show a normal distribution (Figure S1). In contrast, we found that nine out of ten *ppk-1(RNAi)* embryos show reduced GPR-1/2 staining compared to wild-type (Figures 5A and 5B). Similarly, we found that oocytes of *ppk-1(RNAi)* hermaphrodites showed decreased GPR-1/2 levels (Figures S2C and S2D). Thus, PPK-1 positively regulates GPR-1/2.

To determine whether PPK-1 has a role in spindle movements, we performed *ppk-1(RNAi)* in a YFP::tubulin strain and imaged the spindle under a spinning disk microscope. In wild-type embryos, the posterior centrosome displays a rocking movement that is caused by high pulling forces (Grill et al., 2003). Reduced forces in embryos partially depleted for GPR-1/2 causes loss of rocking (Grill et al., 2003; Pecreaux et al., 2006). Consistent with a reduction of spindle forces in *ppk-1(RNAi)* embryos, we found that they showed a decrease in the amplitude of rocking compared to wild-type (Figure 5C). Whereas posterior centrosome movements span 18.4% (±3.2%) of embryo width in wild-type embryos (n = 5), movement spans only 7.1% (±1.7%) in *ppk-1(RNAi)* embryos (n = 6).

To look more directly at pulling forces, we performed spindle severing experiments. We found that peak spindle pole velocities after spindle severing in *ppk-1(RNAi)* are significantly decreased compared to wild-type (Figures 1I and 1J). Therefore, PPK-1 positively regulates GPR-1/2 levels and spindle pulling forces. We deduce that CSNK-1 controls GPR-1/2 and spindle movements through modulation of PPK-1 activity or localization.

### CSNK-1 Regulates PIP_2_ Levels

**Figure 6:**
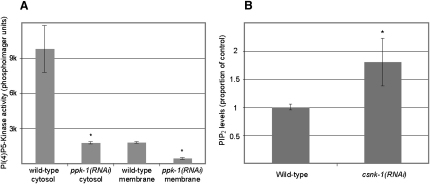
PIP_2_ Levels Are Reduced in *csnk-1(RNAi)* Embryos (A) PI(4)P5-kinase activity in wild-type and *ppk-1(RNAi)* cytosolic and membrane fractions. *ppk-1(RNAi)* extracts have approximately 5-fold less activity. (B) Normalized PIP_2_ mass in wild-type and *csnk-1(RNAi)* embryo extracts. PIP_2_ mass was measured relative to total phospholipids and set to 1.0 for wild-type. *csnk-1(RNAi)* embryos show a 1.8-fold increase in PIP_2_ levels. ^∗^p < 0.01.

Our results support a model whereby CSNK-1 regulates GPR-1/2 and LIN-5 localization at the cortex through negative regulation of PPK-1. PPK-1 is the sole PI(4)P5-kinase in the worm, and its overexpression leads to increased levels of PIP_2_ in vivo (Weinkove et al., 2008). We found that PI(4)P5-kinase activity is strongly reduced in *ppk-1(RNAi)* extracts, confirming that PPK-1 is a PI(4)P5-kinase (Figure 6A). If CSNK-1 negatively regulates PPK-1 in the embryo, then PIP_2_ levels should increase in *csnk-1(RNAi)* embryos. Indeed, we found that *csnk-1(RNAi)* embryos have a 1.8-fold increase of PIP_2_ levels compared to wild-type (Figure 6B, p < 0.01). We conclude that CSNK-1 negatively regulates PIP_2_ production, most likely through negative regulation of PPK-1 localization and/or activity.

## Discussion

In multiple different systems, spindle position and/or orientation during asymmetric cell division is controlled by conserved PAR polarity proteins and their regulation of heterotrimeric G protein activity (reviewed in McCarthy and Goldstein, 2006). We have uncovered a connection between these pathways involving a casein kinase 1 and PI(4)P5-kinase, a PIP_2_ synthesis enzyme.

Gα subunits are key effectors of spindle positioning in animal cells and are regulated by two components which are proposed to form a complex with Gα: a large coiled-coil proposed scaffolding protein (LIN-5 [*C. elegans*], Mud [*Drosophila*], or NuMA [mammals]) and GDP dissociation inhibitors that act as receptor-independent G protein regulators (GPR-1/2 [*C. elegans*], Pins [*Drosophila*], or LGN [mammals]) (reviewed in McCarthy and Goldstein, 2006). How these proteins respond to PAR polarity is unknown in any system. We demonstrate that CSNK-1 regulation of PPK-1 links the conserved PAR and G protein pathways in the control of asymmetric spindle positioning in *C. elegans* by controlling cortical levels of GPR-1/2 and LIN-5.

*csnk-1(RNAi)* embryos have normal PAR polarity but increased levels and loss of asymmetry of GPR-1/2 and LIN-5 at the cortex, causing excessive spindle and pronuclear movements. *ppk-1(RNAi)* embryos show the opposite phenotype: decreased GPR-1/2 and reduced spindle pulling forces. Together with the finding that CSNK-1 inhibits anterior localization of PPK-1 and downregulates PIP_2_ levels, our results indicate that CSNK-1 negatively regulates PPK-1. This is likely to be a direct interaction, because CSNK-1 orthologs of *S. cerevisiae* and *S. pombe* phosphorylate PPK-1 orthologs (Audhya and Emr, 2003; Vancurova et al., 1999).

**Figure 7:**
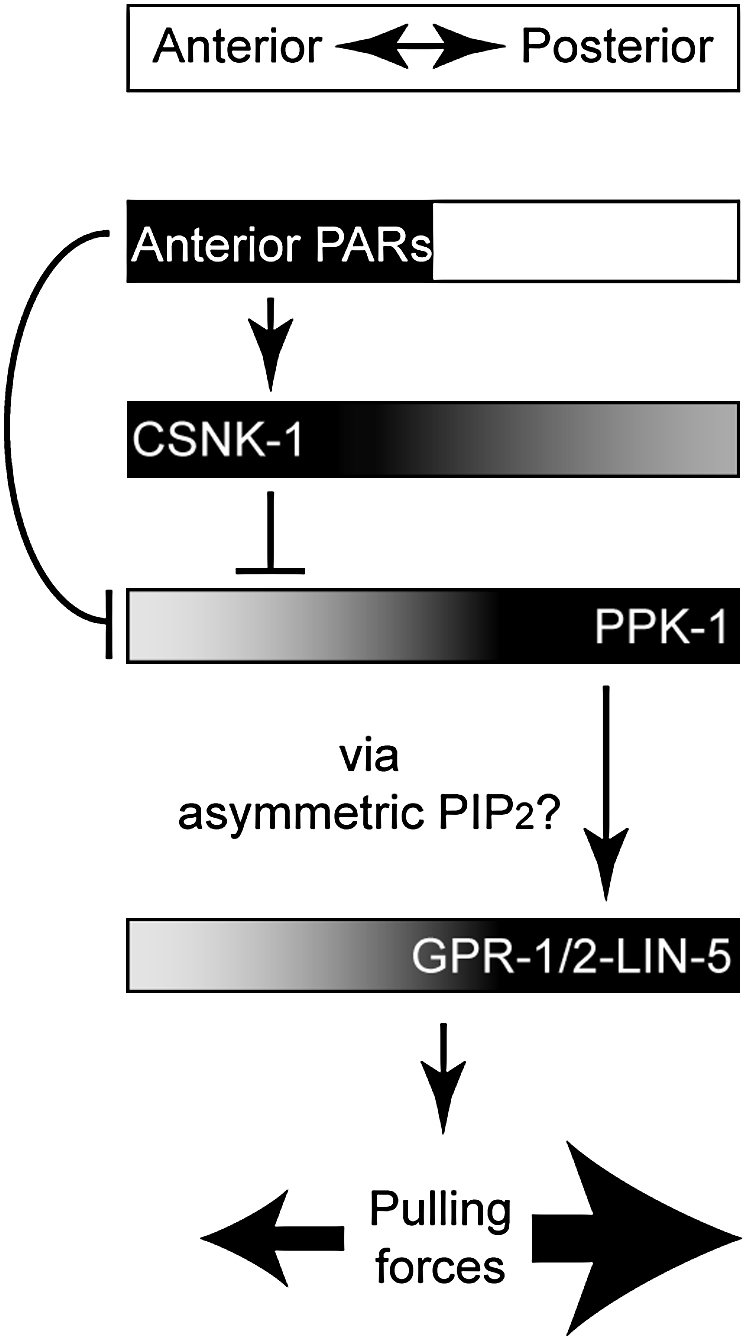
Working Model for CSNK-1 and PPK-1 in Spindle Positioning Proposed distribution and activity of proteins along the A/P axis are indicated by their position in the boxes. Lines with bars indicate antagonistic interactions, whereas lines with arrows depict positive interactions. In this model anterior PAR proteins regulate PPK-1 localization through both CSNK-1-dependent and CSNK-1-independent mechanisms. Posterior enrichment of PPK-1 would lead to asymmetric generation of PIP_2_, which in turn would lead to posterior enrichment of LIN-5 and GPR-1/2 and asymmetric pulling forces.

Our results support a model whereby CSNK-1 links PAR asymmetry to asymmetric forces acting on the spindle by regulating GPR-1/2 and LIN-5 localization at the cortex through PPK-1. The link between PAR polarity and CSNK-1 appears to be via anterior enrichment of CSNK-1. PPK-1 also appears to be regulated by a PAR-dependent but CSNK-1-independent mechanism, since early asymmetry of PPK-1 is disrupted in *par-3* mutant, but not *csnk-1(RNAi)*, embryos (Figure 7).

We propose that enrichment of PPK-1 at the posterior would lead to asymmetric generation of the lipid PIP_2_, which in turn would lead to posterior enrichment, in an unknown manner, of LIN-5 and GPR-1/2 (Figure 7). In the absence of CSNK-1 and its inhibitory role, PPK-1 is uniformly high at the cortex, which would lead to high cortical levels of the lipid PIP_2_, high cortical enrichment of GPR-1/2 and LIN-5, and increased spindle pulling forces. As yet, we do not know what responds to PIP_2_. It is possible that either GPR-1/2 or LIN-5 could bind this lipid, but neither protein has a known PIP_2_-binding domain. Another possibility is that one of these proteins could bind to an as yet unidentified PIP_2_-binding protein. Alternatively, a different phosphoinositide might be more directly relevant for spindle positioning, and interfering with PIP_2_ disrupts its levels. Additionally, despite PPK-1 being a PI(4)P5-kinase, we cannot rule out other models whereby PPK-1 controls spindle positioning by directly binding downstream effectors rather than by producing PIP_2_. A key goal for the future is to identify the mode of action of PPK-1.

### Phosphoinositides and Polarity

Controlled localization of proteins to specific membranes at particular times is critical in the regulation of many intracellular processes. Such localization is often driven by reversible association with particular membrane lipids. To our knowledge, our study is the first showing that asymmetric enrichment of a phosphoinositide synthesis enzyme is important for asymmetric cell division. However, the importance of phosphoinositide asymmetries in polarized events have been described in other systems.

In *Dyctostelium*, in response to chemoattractant concentration, receptor G protein signaling directs PI3-kinases and the lipid phosphatase PTEN to relocate to discrete regions of the membrane that are exposed to higher and lower chemoattractant concentrations, respectively (Devreotes and Janetopoulos, 2003; Funamoto et al., 2002; Iijima et al., 2002). This leads to a gradient of PIP_3_ important for pseudopodia formation (Chen et al., 2003). While the mechanisms of enzyme activation/inhibition have not been established, a similar local accumulation of PIP_3_ controls polarity in other cells, including neutrophils and fibroblasts (Haugh et al., 2000; Wang et al., 2002).

Other studies have described links between the PAR-3 complex and phospohinosotide-generating enzymes. PI3-kinase and PTEN affect the polarization of hippocampal neurons in culture and the localization of PAR-3 and aPKC to the tip of the neurite that is going to become the axon (Jiang et al., 2005; Shi et al., 2003). Recently, it was shown that PTEN directly binds the *Drosophila* PAR-3 homolog, Bazooka (Baz), and colocalizes with it at the apical membrane of epithelia and neuroblasts (von Stein et al., 2005). In *Drosophila* photoreceptors, PTEN is recruited to cell junctions by PAR-3/Bazooka and is important for apical membrane morphogenesis (Pinal et al., 2006). In MDCK cells, PTEN localizes to the apical plasma membrane to mediate the enrichment of PIP_2_, which in turn recruits Annexin2, Cdc42, and aPKC, important for the apicobasal membrane formation (Martin-Belmonte et al., 2007).

Links between phosphoinositide asymmetries and polarity in different organisms and processes suggest widespread roles for phosphoinositides in polarity regulation. In the case of spindle positioning, conservation of involvement of PAR and heterotrimeric G proteins suggests a common transduction mechanism between these pathways. We propose that a central part of such a mechanism involves casein kinase 1 regulation of PI(4)P5-kinases.

## Experimental Procedures

### Strains and Constructs

*C. elegans* worms were handled as described (Brenner, 1974). Strains used in this study were wild-type N2, JA1318 (*Ppie-1: PAR-2-GFP; we9*), JA1354 (*unc-119(e2498); wels12[unc-119(+):pie-1p:GFP:csnk-1*), JA1438 (*dpy-1(e1) par-2(lw32)/sC1*), KK571 (*lon-1(e185) par-3(it71)/qC1 dpy-19(e1259ts) glp-1(q339) III*) (Cheng et al., 1995), and TH65 (*unc-119(ed3) III; Is [Ppie-1:α-tubulin:YFP;unc-119(+)]*) (Schlaitz et al., 2007).

For generation of GFP::CSNK-1 transgenic animals, full-length CSNK-1 was amplified from Y. Kohara cDNA yk610d10 and cloned into pID2.02 (containing *unc-119(+)*, kindly provided by G. Seydoux) according to the manufacturer's protocol (Gateway cloning technology, Invitrogen). The primers used were 5′-GGGGACAAGTTTCTACAAAAAAGCAGGCTTGACGAACACACGCGGGA-3′ and 5′-GGGGACCACTTTGTACAAGAAAGCTGGGTCCTATTTTTGTGTAGCTGGGGTCGCATT-3′. Microparticle bombardment of the plasmid into *unc-119(e2498)* mutants was performed using a Bio-Rad PDS-1000/He according to published protocols (Praitis et al., 2001). This resulted in the integrated strain JA1354 expressing GFP::CSNK-1. We tested whether GFP::CSNK-1 is functional by depleting endogenous CSNK-1 through 3′UTR-directed RNAi of *csnk-1* (see RNA Interference). The GFP::CSNK-1 harbors the *pie-1* 3′UTR and will not be targeted. *csnk-1* 3′UTR RNAi induces 41% lethality in wild-type (n = 87), compared to 8% (n = 60) in GFP::CSNK-1, indicating that the GFP fusion is functional.

### Antibody Production

Antibodies to CSNK-1 were raised against the C-terminal part of the protein (amino acids 312–408) fused to GST and affinity purified using the same fusion protein after depleting the serum of GST antibodies. Antiserum against PPK-1 was raised on two peptides based on the predicted C-terminal sequence of PPK-1 (CGGYRLLKKMEHTWKAILHDGD, CGGSVHNPNFYASRFLTFMTEK), which were synthesized with a 3 amino acid (CGG) N-terminal linker. The peptides were then individually coupled to keyhole limpet hemocyanin and pooled for injection into rabbits. The resulting antiserum was then affinity purified with an MBP-PPK-1 fusion protein.

### Immunofluorescence and Western Blot

Antibody staining was carried out as in Le Bot et al. (2003). The following primary antibodies were used: rat anti-PAR-3 (Dong et al., 2007), mouse anti-LIN-5 (Lorson et al., 2000), chicken anti-GFP (Chemicon), and mouse anti-tubulin antibodies (Sigma, clone DM1A1). Rabbit polyclonal antibodies were used for the following: anti-CSNK-1, anti-PPK-1, anti-GPR-1/2 (Couwenbergs et al., 2004), anti-GOA-1 (Gotta and Ahringer, 2001), and anti-GPA-16 (Afshar et al., 2005). FITC and Texas red secondary antibodies were purchased from Jackson Immunoflourescence. Embryos were imaged either under a Bio-Rad Radiance 2100 Confocal system on a Nikon Eclipse E800 microscope with a Zeiss LaserSharp 2000 Software, or using a Zeiss LSM 510 META system on a Zeiss Axioplan2 microscope. Images were processed using Adobe Photoshop CS2 9.0. Quantification of cortical staining of GPR-1/2 was determined using Image J software. Using Plot Profile, overall fluorescence intensities were obtained from a line drawn all around the cortex starting from the middle of the anterior. For anterior or posterior intensities, a line was drawn around 0%–25% or 75%–100% of egg length, respectively. Quantification of cortical LIN-5 in embryos was determined using the LSM 510 software. Five lines were drawn across the anterior and across the posterior cortices. The peak intensity of each line was recorded and the five numbers averaged to give the final number. Oocyte cortical GPR-1/2 intensity was done in a similar way as for LIN-5, except that peak intensities of the first three cortices were averaged (five values for each cortex). Quantification of cytoplasmic GPR-1/2 oocyte staining was determined by drawing a single line of 10 μm length through the cytoplasm of the most proximal oocyte. This generated 1000 value points, which were averaged to give a single number for each oocyte. SDS-PAGE and western blot analysis were performed according to standard procedures.

### RNA Interference

*csnk-1*, *gpr-1/2*, and *lin-5* RNAi was performed by injection (unless otherwise stated, see below). To prepare dsRNA for RNAi, templates for in vitro transcription were made by performing PCR on bacterial strains (clone sjj_Y106G6E.6 for *csnk-1*, sjj_C38C10.4 for *gpr-2*, and sjj_T09A5.10 for *lin-5*) as described in Kamath et al. (2003) by using T7 primers. 3′UTR of *csnk-1* was amplified from genomic DNA using T7 flanked primers (forward: TAATACGACTCACTATAGGTCTAGTTGCTCACACTGATGC and reverse: TAATACGACTCACTATAGGTAGTGATAGGTGAGAAAAAGTC). dsRNA was in vitro transcribed by using these templates and T7 polymerase (Promega Ribomax RNA production system). dsRNA was injected at a concentration of 0.5–1 mg/ml. Adult hermaphrodites were injected and embryos were analyzed 48 hr after injection at 15°C. For *csnk-1* and *par* epistasis experiments, RNAi was performed by feeding L4 larvae as described in Kamath et al. (2003) for 36 hr at 25°C. For GPR-1/2 staining of *ppk-1(RNAi)* embryos, L4s were fed for 27 hr at 25°C. For PIP_2_ mass assay, L3 larvae were fed on *csnk-1* or L4440 vector dsRNA-expressing bacteria for 48 hr at 20°C.

### Spindle Severing

Spindle serving experiments were performed and analyzed as described in Couwenbergs et al. (2007).

L1 Tubulin::YFP larvae were put on OP50-seeded NGM plates at 25°C and incubated for 27 and 24 hr for *ppk-1(RNAi)* and *csnk-1(RNAi)*, respectively. L3/L4 larvae were washed several times with M9 and put on the seeded 1 mM feeding plates. For *ppk-1(RNAi)*, worms were fed for 27 hr at 25°C and for *csnk-1(RNAi)* for 38 hr at 25°C. As a control the L4440 vector was fed with identical feeding conditions.

### PtdIns(4,5)P_2_ Mass Assay

L3 larvae were fed on *csnk-1*, *ppk-1* or L4440 vector dsRNA-expressing bacteria for 48 hr at 20°C. Embryos were harvested by bleaching (500 mM NaOH, 15% bleach) and transferred into a siliconized eppendorf tube before being frozen on dry ice. Eggs were thawed and resuspended in 200 μl of 2.4 N HCl and were disrupted by sonication. A total of 250 μl of choloform and 500 μl of methanol were added, and the samples were incubated at room temperature for 20 min. The single phase extraction was split by the addition of 250 μl of chloroform and 250 μl of water, and the lower phase (containing the PtdIns(4,5)P_2_) was removed to a clean eppendorf and washed once with theoretical upper phase. The lower phase was dried, and polyphosphoinostides were captured using neomycin affinity chromatography. Neutral lipids, pser and pcho, not bound to the neomycin beads, representing 99% of the extracted phospholipid were used to determine inorganic phosphate levels used to normalize the PtdIns(4,5)P_2_ mass data. Eluted phosphoinositdes from the neomycin column were dried and resuspended in 100 μl of chloroform and 1, 2, and 4 μl were spotted in triplicate onto nitrocellulose filters. A standard curve of known concentrations of PtdIns(4,5)P_2_ were also spotted (50 pmol to 0.375 pmol by serial doubling dilutions). The nitrocellulose was blocked using TBS (pH 7.5)-BSA 1% containing 0.5% (v/v) of Roche western blocking solution after which the blot was probed overnight using GST-PH domain from PLCδ1 (0.2 μg/ml). The blots were washed in TBS, and the interaction of the GST-PH domain was established using an anti-GST antibody and a secondary anti-mouse coupled to horseradish peroxidase. Visualization was carried out using SuperSignal (Pierce Chemicals), and emitted light was captured using a Fuji bas chemiluminescent imager.

PtdIns(4,5)P_2_ was determined from the standard curve, and only values that were in the linear part were used for analysis. The mass of PtdIns(4,5)P_2_ was normalized to the inorganic phosphate obtained from perchlorate digestion of lipids that did not bind to the neomycin column. As the absolute levels of PtdIns(4,5)P_2_ varied among experiments, the data were further normalized to one of the control value samples. The data are plotted as the average data from two separate experiments.

### PI(4)P5-Kinase Activity Assay

Wild-type and *ppk-1(RNAi)* adults were collected and frozen on dry ice. Worms were thawed and sonicated in 200 μl of swell buffer (10 mM Tris [pH 7.5], 1.5 mM KCl, 3 mM MgCl_2_), and membranes were separated from cytosol by centrifugation (14,000 rpm, 10 min). Membranes were washed once with 0.5 ml of swell buffer and finally were resuspended in 200 μl of FRB (10 mM Tris [pH 7.5], 1.5 mM KCl, 3 mM MgCl_2_, 1 mM EGTA, 0.32 M sucrose). Protein concentration was determined using Biorad protein assay reagent, and 10 μg was used for PtdIns(4)P5-kinase activity measurements, while 20 μg was used for western blotting to confirm knockdwn of PPK-1. For the PtdIns(4)P5-kinase activity assay, 10 μg of cytosol protein was incubated together with lipid substrate (0.5 nmol PtdIns4P and 10 nmol PtdSer), while membranes were incubated in the absence of added substrate in 90 μl of FRB. Reactions were initiated by the addition of 10 μl of FRB containing 10 μM ATP and 5 μCi [^32^P]ATP. Reactions were carried out for 5 min, after which labeled lipids were extracted and analyzed by thin-layer chromatography. Incorporation of ^32^P into PtdIns(4,5)P_2_ was monitored using a phosphoimager (Biorad).

### DIC and GFP Movies

Live imaging of embryos was performed as described (Zipperlen et al., 2001) using Improvision Openlab software. For Figures 1A–1F, eight focal planes were taken every 10 s. Movies using GFP or YFP strains were performed using a Perkin-Elmer spinning disk confocal system. Images were taken every second in a single focal plane and processed using Adobe Photoshop CS2 9.0.

## Supplemental Data

Document S1. Five Supplemental Figures

Movie S1. GFP:CSNK-1 Dynamics Signal is detected at the membrane and in cytoplasmic puncta. The puncta appear to form by internalization at the plasma membrane and move towards the asters.
