# Supplementary material for: A Casein Kinase 1 and PAR Proteins Regulate Asymmetry of a PIP2 Synthesis Enzyme for Asymmetric Spindle Positioning
Source: Dev Cell. 2008 Aug 12;15(2):198–208. doi: 10.1016/j.devcel.2008.06.002 (PMC2686839; doi:10.1016/j.devcel.2008.06.002)
Supplement: Document S1. Five Supplemental Figures [file mmc1.pdf]

Developmental Cell *15*

Supplemental Data

A Casein Kinase 1 and PAR Proteins Regulate Asymmetry of a PIP_2 Synthesis Enzyme for Asymmetric Spindle Positioning

Costanza Panbianco, David Weinkove, Esther Zanin, David Jones, Nullin Divecha, Monica Gotta, and Julie Ahringer

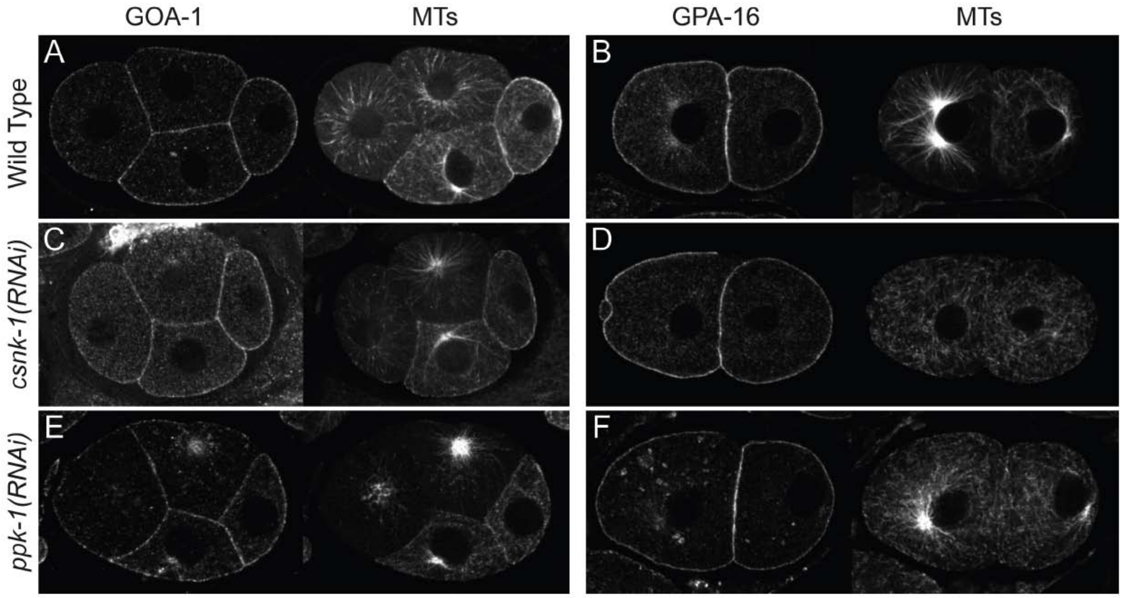

Figure S1. GOA-1 and GPA-16 Distribution Is Unaltered in *csnk-1(RNAi)* and *ppk-1(RNAi)* Embryos
Wild-type (A, B), *csnk-1(RNAi)* (C, D) and *ppk-1(RNAi)* (E, F) embryos stained for GOA-1 (A, C, and E-left panels) and GPA-16 (B, D, and F-left panels). Tubulin is shown in the right panels.

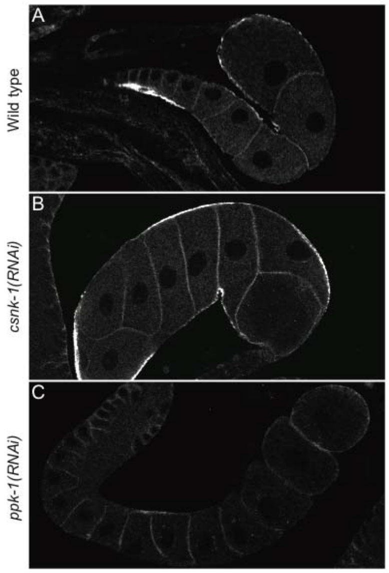

D

	Cortical	Cytoplasmic
**Wild type**	74 (± 3); n=33	26 (± 1.9); n=13
csnk-1(RNAi)	97 (± 5.4); n=29	27 (± 3.3); n=12
ppk-1(RNAi)	56 (± 2.9); n=39	14 (± 1.1); n=14

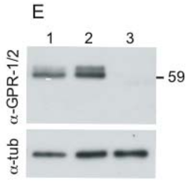

Figure S2. Cortical GPR-1/2 Is Increased in *csnk-1(RNAi)* and Decreased in *ppk-1(RNAi)* Gonads

(A–C) Dissected gonads of (A) wild-type (B) *csnk-1(RNAi)*, and (C) *ppk-1(RNAi)* adult hermaphrodites stained for GPR-1/2. Cortical GPR-1/2 is higher in (B) *csnk-1(RNAi)* gonad and lower in (C) *ppk-1(RNAi)* gonad compared to (A) wild-type gonad.

(D) Quantification of cortical and cytoplasmic GPR-1/2 pixel intensity in wild-type, *csnk-1(RNAi)* and *ppk-1(RNAi)* gonads (mean ± SEM). For measurement of cortical intensity, the average peak intensity of the three most proximal oocyte cortices is given. n, number of individual cortices. Cytoplasmic number gives the average pixel intensity of the most proximal oocyte. n, number of oocytes analysed.

(E) Western blot of wild-type (lane 1), *csnk-1(RNAi)* (lane 2) and *gpr-1/2(RNAi)* (lane 3) embryo extracts, probed with anti-GPR-1/2 (top) or alpha-tubulin loading control (bottom). Quantification was carried out using Gels Analyze tool in Image J. Ratio of GPR-1/2 to tubulin in wild type was normalised to 1. In *csnk-1(RNAi)* embryos, this ratio is 0.96 indicating no difference in GPR-1/2 levels.

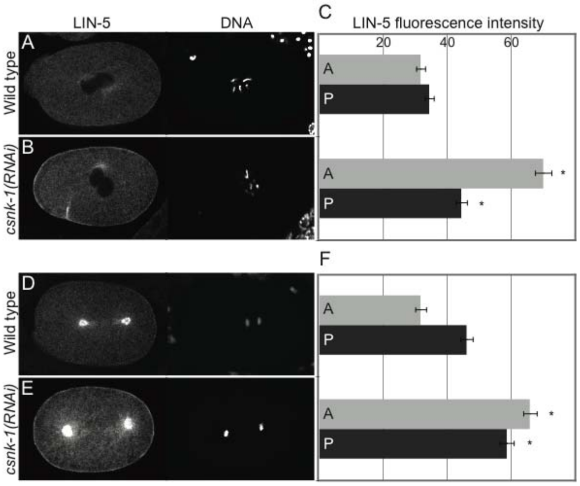

Figure S3. Increased Cortical LIN-5 in *csnk-1(RNAi)* Embryos
(A, D) Wild-type and (B, E) *csnk-1(RNAi)* embryos stained for LIN-5 (left panels) and DNA (right panels). (A, B) One-cell embryos during centration. (D, E) One-cell embryos at anaphase. LIN-5 is weakly enriched at the posterior in wild-type anaphase embryos (D). *csnk-1(RNAi)* embryos show increased cortical LIN-5 (B, E), anterior enrichment of LIN-5 during centration (B) and no asymmetry in anaphase (E). (C, F) Average anterior (light grey) and posterior (dark grey) cortical LIN-5 pixel intensities in specified genotypes of embryos (C) at pronuclear meeting and (F) during meta to telophase (bar represents SEM).
* indicates p value < 0.01 compared to corresponding wild-type.

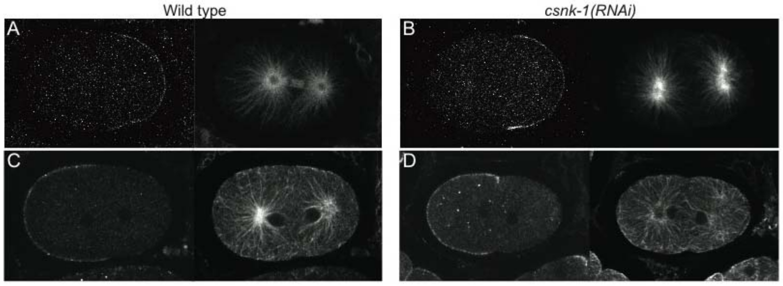

Figure S4. *csnk-1(RNAi)* Embryos Have Normal PAR Polarity
Wild-type (A, C) and *csnk-1(RNAi)* (B, D) embryos stained for PAR-2 (A, B left panels) and PAR-3 (C, D left panels). Tubulin is shown in the right panel for each image.

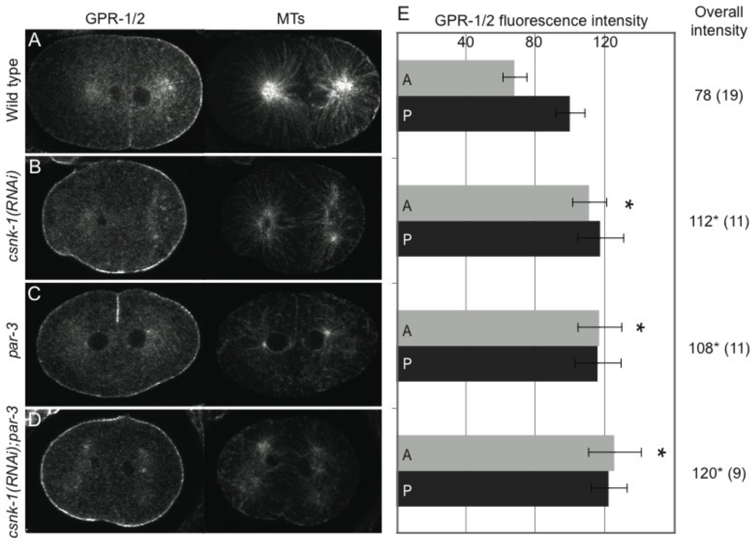

Figure S5. GPR-1/2 Distribution Depends on CSNK-1 and PAR-3

GPR-1/2 (left panels) and tubulin (right panels) staining in embryos of indicated genotypes.

(A-D) One-cell embryos at telophase.

(E) Average anterior cortical (0-25%; light grey) and posterior cortical (75-100%; dark grey) GPR-1/2 pixel intensities in specified genotypes of embryos at during metaphase to telophase (bar represents SEM).

* indicates p value < 0.01. Overall intensity signifies the average intensities of whole embryonic cortices.
